# Factors related to medication errors in the preparation and administration of intravenous medication in the hospital environment

**DOI:** 10.1371/journal.pone.0220001

**Published:** 2019-07-24

**Authors:** Verónica V. Márquez-Hernández, Ana Luisa Fuentes-Colmenero, Felipe Cañadas-Núñez, Marco Di Muzio, Noemi Giannetta, Lorena Gutiérrez-Puertas

**Affiliations:** 1 Department of Nursing, Physiotherapy and Medicine, Faculty of Health Sciences, University of Almería, Almería, Spain; 2 Research Group for Health Sciences, University of Almería, Almería Spain; 3 Hospital Universitario Torrecárdenas, Almería, Spain; 4 Department of Clinical and Molecular Medicine, Sapienza University of Rome, Rome, Italy; 5 Department of Biomedicine and Prevention, Tor Vergata University of Rome, Rome, Italy; Nord University, NORWAY

## Abstract

**Background:**

Medication errors have long been associated with low-quality medical care services and significant additional medical costs.

**Objective:**

The aim of this study was to culturally adapt and validate the questionnaire on knowledge, attitudes and behaviors in the administration of intravenous medication, as well as to explore these factors in a hospital setting.

**Methods:**

The study was divided into two phases: 1) validation and cross-cultural adaptation, and 2) cross-sectional study. A total of 276 hospital-based nursing professionals participated in the study.

**Results:**

A Cronbach’s alpha value of 0.849 was found, indicating good internal consistency. In the multivariate analysis, statistically significant differences were found between knowledge and attitudes, demonstrating that having greater suitable knowledge correlates with having a more positive attitude. It was also discovered that having a positive attitude as well as the necessary knowledge increases the possibility of engaging in adequate behaviors.

**Conclusions:**

The knowledge, attitudes and behavior questionnaire has a satisfactory internal consistency in order to be applied to the Spanish context.

Implications for nursing management: Knowledge acquisition and positive attitude are both factors which promote adequate behavior, which in turn seems to have an impact on medication errors prevention. Health institutions must encourage continuous education for their employees.

## Introduction

Medication errors (MEs) have long been related to low-quality medical care services [1], longer hospital stays [2], significant additional medical costs [3], as well as loss of patient trust in the services offered by the hospital [4]. According to the National Coordinating Council for Medication Error Reporting and Prevention (NCCMERP), a medication error is any preventable event that may cause or lead to improper medication use or patient harm while the medication is under the control of a healthcare professional, patient or consumer [5].

Estimating the prevalence of MEs is problematic, due to the different definitions and classification systems that are typically used to measure them. Consequently, study results may have a high degree of variability, anywhere from 2% to 94% [6]. In the case of Spain, there are approximately 17 medication errors a day per 100 hospitalized patients, 16% of which are related to prescription, 27% transcription, 48% in dispensation, and finally, 9% in administration [7].

Many authors have investigated the reasons behind MEs, finding that they may be due to numerous factors within the system, as well as individual factors [8,9]. Some of the main causes of MEs are dosage calculation errors [10], overworked hospital units [11–13], employee fatigue [14], insufficient knowledge [15,16], and unsuitable environmental conditions [17]. MEs can happen at any stage [18], but it is during the phases of preparation and administration of medicine when there is a greater possibility of such errors occurring [19–21]. In addition, studies have observed that MEs can be related to the administration method. More specifically, a prospective observational study found that nearly 70% of medications administered intravenously (IVs) involved a clinical error [22]. IV medications present particular risks due to their greater complexity and the multiple steps required for their preparation, administration and monitoring [22–24].

In the clinical setting, nurses are responsible for setting up and administering most medicines directly to patients. Although providing medication is a multidisciplinary process, nurses take on almost all the responsibility for administering and controlling the medication used [25]. It is estimated that 78% of nurses have committed a medication error at some point [26], and it was established that 40% of their clinical time is devoted to managing medication [27]. This translates into approximately 16 hours of a nurse's working week being dedicated to handling medicines [28].

There are many different studies that have focused on the reporting of MEs by nursing professionals [9,29,30], however, there is a lack of research concerning the knowledge, attitudes and behaviors of nursing professionals in the preparation and administration of IVs [4,31,32]. With this in mind, Di Muzio, Tartaglini, De Vito and La Torre [32] developed a questionnaire in order to measure the knowledge and attitudes of nurses in the preparation of medication.

Nonetheless, in Spain, there are no real tools that exist to measure the knowledge, attitudes and behaviors of nurses during IV medication preparation and administration phases, nor studies which examine the study of these variables at a hospital level in more depth. The objective of this study was thus to culturally adapt and validate the knowledge, attitudes and behavior questionnaire for the administration of IV medicine, as well as to explore these three factors in a hospital environment.

## Methods

### Design

The study was divided into two phases: 1) validation and cross-cultural adaptation and 2) cross-sectional study.

### Sample

The study was conducted at the Torrecardenas University Hospital (Almeria. Spain) from April to June 2018. Out of the total possible number of participants (N = 817), 276 nurses participated who administered IV medication daily. The sample was selected through a convenience sample. The recommendation of 5–10 subjects per questionnaire item was considered to analyze the psychometric properties of the assessment [33]. The inclusion criteria were as follows: nurses who worked in the hospital of study, who, throughout their workday, administered IV medicine, and who could speak and read Spanish. The exclusion criteria were as follows: nursing professionals with short-term contracts, as well as any subject who chose not to participate.

### Measurements

The knowledge, attitudes and behavior (KAB) questionnaire was developed by Di Muzio et al. [32]. This questionnaire consists of 19 questions, or items, divided into three dimensions: knowledge (7 questions), attitudes (7 questions) and behavior (5 questions). In addition, the questionnaire included questions about the participants’ socio-demographic and professional characteristics, access to up-to-date information, and the need for continuous education related to the use of IV medication. The original authors found a Cronbach’s alpha value of 0.776, indicating satisfactory internal validity.

The questions concerning the dimensions of knowledge and behavior were scored on a Likert-type scale with 5 response options (disagree, somewhat agree, moderately agree, mostly agree, totally agree). In contrast, a Likert-type scale of 3 possible responses (agree, uncertain and disagree) was provided to answer the questions for the attitudes dimension.

### Procedures for data collection

#### Item generation

To initiate the cultural adaptation process, a back-translation protocol was followed, based on the standardized recommendations detailed by Beaton, Bombardier, Guillemin and Ferraz [34]. Two separate simultaneous translations were completed by two bilingual professionals, whose mother tongue was English. The versions obtained through the translations were revised, compared and analyzed in a systematic way, checking each of the items on the translated scales. Any unusual or confusing concepts were discussed by members of the research team and the translators, and a first version of the scale in Spanish was obtained. From this first version, a back-translation was done back into the original language by two native English speakers. This back-translated version was compared with the original version in order to detect any discrepancies.

#### Content validity analysis

The consensual version was revised by a panel of experts, composing of 14 nurses (supervisors, university professors, or clinical tutors), who assessed each item as either unnecessary, useful, or essential, in order to later calculate the Content Validity Index (CVI), which is described in depth in the Results section. Consequently, a final version was tested on 20 professionals to evaluate the intelligibility of the items. In order to validate the content, the adapted consensus version of the KAB questionnaire was evaluated by a panel of experts, obtaining the definitive version, as previously explained.

#### Construct validity and reliability

In terms of structural validity and reliability, the necessary analyses were performed, which are described in the Results section. Once permission was obtained, the main researcher contacted the nursing supervisors of each clinical unit, in order to explain the objective of the study and ask for their compliance in recruiting professionals from their unit.

### Ethics statement

Prior to data collection, the main researcher asked for permission from the Research Ethics Committee, in this case known as the Research Ethics Committee of Almeria (CEIC) (20/2018), which confirmed that the study respected the ethical principles outlined in the Declaration of Helsinki in 2013 and other International Codes. All the participants gave their written informed consent before commencing participation in the study. In addition, each professional participant was informed of the voluntary nature of their participation, as well as the possibility to opt out of the study at any time. They were also informed of the anonymity and confidentiality of their data.

### Data analysis

The statistical program SPSS version 20 was used for data analysis. First, a descriptive statistical analysis was performed on the participants' socio-demographic data, calculating measures of central tendency and dispersion (mean and standard deviation) for the quantitative variables, while frequencies and percentages were calculated for the categorical variables. The CVI proposed by Lawshe [35] was used to calculate the content validity index. A confirmatory factorial analysis was performed.

Additionally, the Kaiser-Meyer-Olkin (KMO) test was used to check sample suitability, whereas Cronbach’s alpha was calculated to analyze the internal consistency. For the bivariate analysis, the chi squared test was used for categorical variables. In contrasting the hypothesis between quantitative and qualitative variables, in which the latter did not follow a normal distribution, the non-parametric Mann-Whitney U test was used. A value of p<0.05 was considered significant. Finally, three multiple logistic regression models were developed, with the purpose of identifying the predictors of knowledge, attitudes and behaviors, following the model established by Di Muzio et al. [4].

## Results

### Sample demographic characteristics

A total of 276 nursing professionals took part in the study, of which 74.6% (n = 206) were female and 25.3% (n = 70) were male. The average age was 45.00±7.59 years, with a range between 21 and 63 years old. Additional information on the socio-demographic and professional characteristics of the participants is displayed in Table 1.

**Table 1:** Participants' socio-demographic and professional characteristics.

Variable	n	%
***Demographic Characteristics***		
Sex:		
Male	70	25.3
Female	206	74.6
Age	45.00*	7.59**
***Professional Characteristics***		
Years of Experience:	21.69*	7.77**
Postgraduate education:		
Master	57	20.7
Others	88	31.9
None	131	47.5
Undergraduate education related to the preparation and administration of IV medicine:		
No	42	15.2
Yes	216	80.3
***Continuous professional development***		
Internet access at the workplace:		
No	99	35.9
Yes	177	64.1
Library (also online) available at the workplace:		
No	114	41.3
Yes	162	58.7
Hours dedicated to continuous health education:		
<1h/week	133	48.2
1-5h/week	119	43.1
6-10h/week	18	6.5
>10h/week	6	2.2

*Mean

**Standard Deviation

Regarding the need for proper training, 80.8% of the participants reported having a good level of knowledge on the preparation and administration of IV medicine, although they considered it essential to improve their skills (88.1%).

### Phase I: Validation and cross-cultural adaptation

Firstly, for the transcultural adaptation of the questionnaire, the protocol described previously in the Methods section was used. The CVI was calculated by the panel of experts, and 2 questions were eliminated from the knowledge dimension since they did not have a CVI value above 0.80. The questions that were eliminated were “Availability of informative protocols, posters and brochures in the wards, promotes the decrease of the error risk” and “Assistance of a pharmacist during drug preparation reduces the error risk”.

Regarding the psychometric properties, the KAB questionnaire had a Cronbach’s alpha value of 0.849, indicating good internal consistency [34]. In addition, Cronbach’s alpha was calculated again after certain items were deleted, with values being found in a range between 0.834–0.855, which showed no benefit in deleting any of the items (Table 2).

**Table 2:** Cronbach’s alpha if one of the items was deleted.

Item	Scale mean if the item was deleted	Variance in scale if the item was deleted	Corrected total correlation of the items	Cronbach’s alpha if the item was deleted
Knowledge: Item 1	48.40	29.333	.343	.849
Knowledge: Item 2	48.90	27.092	.481	.844
Knowledge: Item 3	48.76	27.746	.555	.836
Knowledge: Item 4	48.90	27.598	.368	.855
Knowledge: Item 5	48.64	27.872	.476	.842
Attitudes: Item 1	50.16	31.071	.724	.842
Attitudes: Item 2	50.18	31.018	.610	.842
Attitudes: Item 3	50.27	31.108	.309	.847
Attitudes: Item 4	50.28	30.424	.479	.842
Attitudes: Item 5	50.23	30.866	.501	.842
Attitudes: Item 6	50.38	30.594	.360	.845
Attitudes: Item 7	50.20	31.091	.516	.843
Behavior: Item 1	48.33	28.717	.625	.834
Behavior: Item 2	48.32	28.837	.646	.833
Behavior: Item 3	48.27	29.114	.637	.834
Behavior: Item 4	48.24	29.206	.665	.834
Behavior: Item 5	48.40	28.500	.616	.834

To validate the content, the adapted version of the KAB questionnaire was evaluated by a panel of experts, obtaining the definitive version, as previously detailed. Contrastingly, for the analysis of the structural validity of the questionnaire, a factorial analysis was performed. The KMO test reported a value of 0.867, above the accepted minimum of 0.70 [36]. The analysis factor was checked for suitability using Bartlett's sphericity test (X^2^(171) = 2146.118; p<0.05). The structure of three factors (knowledge, attitudes and behavior) was then confirmed.

### Phase II: Cross-sectional study

Regarding the knowledge dimension, most participants (82.2%) completely agreed that knowledge of IV medicine dosage calculation could reduce the number of medication errors. In addition, almost half of the participants (48.2%) thought that computerized physician order entry systems (CPOE) could reduce errors during the medicine preparation phase. A similar percentage (48.9%) of the participants stated that the unit dose dispensing system from the pharmacy helped to reduce the number of MEs. More than half the professionals specified that MEs can be caused by disruptions and a heavy workload. Additionally, the youngest participants, and therefore the ones with less experience, stated that knowledge of medicine dose calculation was important; (U = 213.500; Z = -2.374; p = 0.018) and (U = 145.000; Z = -2.833; p = 0.005) respectively. There were no statistically significant differences found among the rest of the socio-demographic variables and the knowledge dimension.

In the multivariate analysis, regarding Model 1 (Knowledge of the administration of IV drugs can reduce medication errors), no statistically significant differences were found in terms of education level, although it was determined that those who had higher education degrees were more knowledgeable. Statistically significant differences were not found between the hospital units and knowledge (Table 3).

**Table 3:** Prevention of medication errors based on nursing professionals' knowledge, attitudes and behaviors.

Variable	OR*	95% CI**
*Model 1*: *Knowledge of the administration of IV drugs can reduce medication errors*.		
Education level	1.13	0.80–1.60
Unit (General Medicine/Emergency and Intensive Care)	1.14	0.92–1.41
*Model 2*: *Attitudes toward medication therapy can predict errors*.		
Hospital Unit	1.07	0.63–1.81
Sex	1.06	0.59–1.88
Preparation and administration of IV medication in undergraduate	0.98	0.50–1.91
Preparation and administration of IV medication in postgraduate	0.51	0.27–0.96
Knowledge	2.51	1.52–4.14
*Model 3*: *Behavior toward medication therapy can predict errors*.		
Education level	0.45	0.12–1.68
Hospital Unit	1.18	0.60–2.02
Postgraduate Education	1.15	0.57–2.34
Knowledge	1.89	1.14–3.12
Attitudes	3.74	2.24–6.26

*Odds Ratio

**Confidence Interval

In terms of attitudes, most participants (96.3%) agreed that specific and continuous training on IV medicine was necessary, as well as better management of clinical risks (92.4%). 87% of the participants stated that motivation is essential to improve workplace performance. The use of protocols, as well as the evaluation of clinical skills, were also considered fundamental, (87%) and (74.6%) respectively. Concerning the reporting of MEs, 90.2% agreed that it should always be done. In the multivariate analysis, regarding Model 2 (Attitudes toward medication management can predict errors), no positive associations were found between attitudes and the hospital unit, sex, or level of education. Nonetheless, a statistically significant increase in positive attitudes was found in those participants who had attended postgraduate courses related to the preparation and administration of IV medicine (OR 0.51, 95% CI 0.27–96) (Table 3). There were also statistically significant differences found between knowledge and attitude (X^2^ (1) = 13.269, p<0.05), demonstrating that greater knowledge leads to more positive attitudes.

Lastly, regarding behavior, most participants strongly agreed that hand washing (82.6%), checking vital signs (82.2%), respecting the infusion rate (87%), following the 5 rights rule (89.9) and double-checking (76.8%) were important factors in reducing complications. In the multivariate analysis, regarding Model 3 (Behavior towards drug therapy can predict errors), no statistically significant correlations were found between educational level, postgraduate training or unit. However, statistically significant differences were found between behavior and knowledge (X^2^(1) = 6.325, p = 0.012) and behavior and attitude (X^2^(1) = 26.609, p<0.05), demonstrating that positive attitudes (OR 3.74, 95% CI 2.24–6.26) and having suitable knowledge (OR 1.89, 95% CI 1.14–3.12) increase the likelihood of adequate behavior (Table 3).

## Discussion

The objective of this study was to culturally adapt and validate the questionnaire on knowledge, attitudes and behavior for the administration of IV medication, as well as explore these three factors in a hospital setting. Initially, regarding the adaptation and validation of the questionnaire, in this study a Cronbach’s alpha value of 0.849 was obtained, which is a slightly higher figure than that found by the two original authors (alpha = 0.776) [32], and both cases have shown satisfactory internal validity.

Firstly, regarding the professional characteristics of the participants, no statistically significant differences were found between the nursing units. However, some studies maintain that there may be a higher number of MEs in hospital units where many complex medication administration activities take place [25], partly due to the different distractions and disruptions that occur there [37], as may be the case in intensive care units [38]. Conversely, no statistically significant differences were found in terms of professional experience. However, studies such as the one conducted by Sears, O´Brien-Pallas, Stevens and Murphy [39], stated that units with more experienced nurses reported their MEs more frequently, which may mean that involving experienced nurses reported their MEs more frequently, which may mean that involving experienced nurses could increase the voluntary reporting of such errors [40].

In the second phase of the study, concerning knowledge, most of the participants strongly agreed that knowledge of IV dosage calculation can reduce errors. Similar findings were described by Di Muzio et al. [4]. In contrast, certain studies indicate that there is insufficient evidence to declare that MEs are caused by nursing professionals' poor mathematical skills [41].

On the other hand, almost half of the participants in this study agreed that CPOE can reduce errors during the preparation of medication. The adoption of CPOE has been shown to reduce MEs and patient harm [42], although, according to the last systematic revision of CPOE, the reporting of prescription errors must continue because the weaknesses of CPOE systems themselves are possible sources of error [43].

Regarding work overload, half of the nursing professionals indicated that this could affect MEs. Higher percentages were found by Di Muzio et al. [4] and various studies state that one of the influential factors for medication errors is an overly heavy workload in hospital units such as the emergency unit [11,13]. This may be due to nurses with heavy workloads not being as attentive to patient safety principles during the provision of medicine which ultimately increases the risk of MEs [44–46].

Considering attitudes, almost all the participants (96.3%) agreed that it was necessary to have specific and continuous training on IV medication. Similarly, Di Muzio et al. [4] found that almost 86% of nurses agreed with the above statement. Regarding undergraduate education, no significant results were found. Nonetheless, it is important to emphasize that nurses should be taught during their undergraduate studies about the possible negative consequences of not reporting MEs [47]. The importance of educational interventions and the role of interdisciplinary teams should also be highlighted, as reported in other studies on residents [48].

Regarding postgraduate education, there was an increase in positive attitudes in those participants who had undertaken postgraduate courses related to the preparation and administration of IV medicine, highlighting the importance of continuous education. A positive association was also found between knowledge and attitudes, which demonstrating that greater knowledge corresponds with a more positive attitude, an association that is not corroborated by the original authors of the questionnaire [4].

In terms of behavior, the interviewees of this study agreed on the importance of basic procedures such as hand washing, following the 5 rights rule, double-checking, etc. In various cases, it has been advocated that nurses double-check before administering high-alert drugs [49]. In addition, the importance of patient identification is stressed, as a significant correlation has been found between not verifying a patient's identity and committing an IV administration error [20]. Finally, it was found that positive attitudes and suitable knowledge increase the likelihood of adequate behavior.

Nevertheless, the results of this study are not without limitations. Firstly, the study is focused on a specific health institution, limiting the possibility of generalizing results. Furthermore, although the sample size was adequate for the validation of the questionnaire according to the literature consulted, future research should use a larger sample size. Lastly, although the results of the psychometric properties were satisfactory, they should be analyzed in other health contexts where IV medication is also used, such as nursing homes. However, there as yet no other tools validated in the Spanish context that can be used to measure the knowledge, attitudes and behavior of nursing professionals in the preparation of IV medication, making this both a pioneering and necessary study in the field, enabling deeper knowledge of the factors that cause MEs.

## Conclusion

Nursing professionals bear most of the responsibility for the preparation and administration of IV medicine. This study shows that the KAB questionnaire has sufficient satisfactory internal consistency to be applied to our context. In addition, the exploration of factors such as knowledge, attitudes and behavior of nursing professionals, allows in-depth insight into the process of IV therapy administration by nurses.
